# Effects of specific nutrients on tax-dependent activation of NF-κB and MMP-9 in human T-cell lymphotropic virus -1 positive malignant T-lymphocytes

**DOI:** 10.1186/1471-2164-15-S2-P72

**Published:** 2014-04-02

**Authors:** Steve Harakeh, Mona Diab-Assaf, Rania Azar, Esam Azhar, Ghazi A Damanhouri, Adeel Chaudary, Haitham Yacoub, Mourad Assidi, Muhammad M Abu-Elmagd, Mohammed H Alqahtani, Adel M Abuzenadah, Taha Kumosani, Aleksandra Niedzwiecki, Mathias Rath, Elie Barbour

**Affiliations:** 1 Special Infectious Agents Unit, King Fahd Medical Research Center, King Abdulaziz University, Jeddah, Kingdom of Saudi Arabia; 2 Molecular Tumorigenesis and Anticancer Pharmacology, Lebanese University, Hadath, Lebanon; 3 Department of Medical Laboratory technology, Faculty of Applied Medical Sciences, King Abdulaziz University, Jeddah, Kingdom of Saudi Arabia; 4 Biological Sciences Department, Faculty of Sciences, King Abdulaziz University, Jeddah, Kingdom of Saudi Arabia; 5 Center of Excellence in Genomic Medicine Research, King Abdulaziz University, Jeddah, Kingdom of Saudi Arabia; 6 Dr. Rath Research Institute, Santa Clara, CA, USA; 7 Department of Animal and Veterinary Sciences, American University of Beirut, Lebanon

## Background

Adult T-cell Leukemia (ATL) is a disease with no known cure so farand it is a resistant to chemotherapy. The virus can be transmitted by exchange of bodily fluids through the placenta and from mother to child. Only 5% of those who are infected develop the disease after a long latency period ranging from 30-50 years [1,2]. The disease manifests itself as an aggressive proliferation of CD4^+^ cells with the human T-cell Lymphotropic virus type 1 (HTLV-1) [3]. The leukemogenesis of the virus is mainly attributed to the viral oncoprotein, Tax, that activates the Nuclear Factor kappa B (NF-κB)which in turn stimulates the activity and expression of the matrix metalloproteinase-9 (MMP-9)which is important in angiogenesis[2]. Our previous work has shown that using non-cytotoxic concentrations of a Specific Nutrient Synergy (SNS)mixture resulted in the induction of apoptosis in both HTLV-1 positive and negative malignant T-lymphocytes [1]. The objective of this study is to investigate the efficacy of SNS on Tax expression, NF-κB levels as well as on MMP-9 activity and expression both at the transcriptional and translational levels intwo HTLV-1 positive cell lines, HuT-102 and C91-PL.

## Materials and methods

Cell growth, experimental design, source of SNS, preparation and storage of stock solution, were previously described by our group [1].The effects of non-cytotoxic concentrations of SNS ranging from 0-350 μg/ml were evaluated for their efficacy on proliferation, Tax expression, NF-κBmobility and the activity and expression of MMP-9 at 48h and 96hof incubation.Cytotoxicity of EGCG was assayed using CytoTox 96 Non-radioactive and proliferation was measured using Cell Titer96^TM^ Nonradioactive Cell Proliferation kit( MTT- based assay). Elisa and EMSA were used to assess the effect of SNS on NF-κBmobility. Zymography was used to determine the effects of SNS on the activity and secretion of MMP-9. The expression of MMP-9 was done using RT-PCR at the translational level and Immunoblottingat the transcriptional level.

## Results

**Figure 1:**
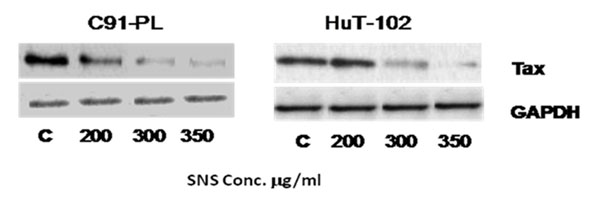
Effect of SNS on Tax expression in C91-PL and HuT-102 cell lines. Equal loading was ensured using GAPDH. The immunoblots represent results obtained in one of three independent experiments.

**Figure 2:**
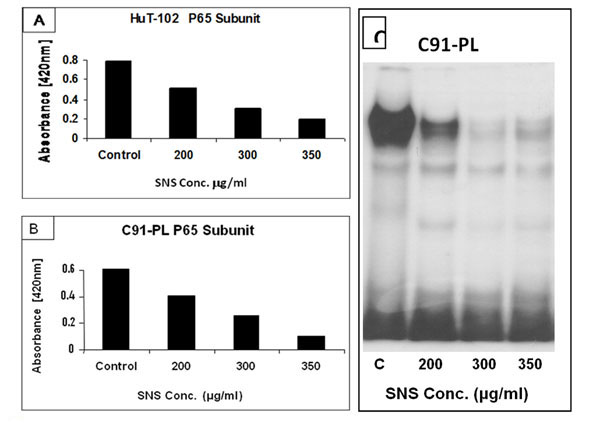
Effect of SNS on NF-κB nuclear translocation in HuT-102 and C91-PL HTLV-1 positive cell lines. (A,B) Nuclear levels of P65 subunit of NF-κB was evaluated by ELISA at 48 and 96 h of incubation. Each value is the mean ± SD deduced from three separate experiments done in triplicate. (C) EMSA Gel representing one of three independent experiments with nuclear extracts of C91-PL.

**Figure 3:**
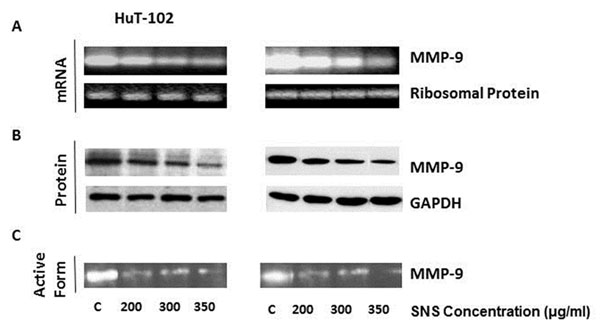
Effect of SNS on MMP-9 mRNA (a), protein (b) and activity (c) in two ATL-HTLV-1 positive cell lines. Equal loading was ensured using ribosomal protein for mRNA expression (a) and GAPDH for protein expression (b).The results represent one out of three independent experiments.

A significantinhibition of proliferation was seen in both cell lines starting at a concentration of 200μg/ml and in a dose dependent manner. SNS induced a dose dependent decrease in Tax expression (Fig.1),which was paralleled by a down-regulation of the nuclearization of NF-κB (Fig.2). This culminated in the inhibition of the activity of MMP-9 and their expression both at the transcriptional and translational levels (Fig.3).

## Conclusions

The role of nutrients in the treatment of disease has been overlooked for a long time. Recently, it has been recognized that nutrients play a crucial role in the outcome of the treatment. The results of this study indicate that a specific nutrient synergy targeted multiple levels pertinent to the progression of ATL. Its activity was mediated through the NF-**κ**B pathway, and hence has the potential to be integrated in the treatment of this disease as a natural, yet potent anticancer agent.
